# A Variant of sNASP Exacerbates Lymphocyte Subset Disorder and Nephritis in a Spontaneous Lupus Model Sle1.Yaa Mouse

**DOI:** 10.1155/2021/8175863

**Published:** 2021-10-21

**Authors:** Jianye Zhang, Xiaoping Du, Hui Wang, Yatao Bao, Meng Lian, Zhiwei Xu, Jiyu Ju

**Affiliations:** ^1^School of Basic Medical Science, Weifang Medical University, Weifang 261053, China; ^2^Medical Control Office, The Second Affiliated Hospital of Weifang Medical University, Weifang 261041, China; ^3^Medical Control Office, Weifang No. 2 Hospital, Weifang 261041, China

## Abstract

A variant of somatic nuclear autoantigenic sperm protein (sNASP) was identified from the murine lupus susceptibility locus Sle2c1 by whole exome sequencing (WES). Previous studies have shown that mutant sNASP could synergize with the Fas^lpr^ mutation in exacerbating autoimmunity and aggravating end-organ inflammation. In the current study, the sNASP mutation was introduced into Sle1.Yaa mice to detect whether it has a synergistic effect with Sle1 or Yaa loci. As expected, compared with Sle1.Yaa mice, Sle1.Yaa.*Δ*sNASP mice showed enlarged lymph nodes, aggravated renal inflammation, and shortened survival time. The proportions of CD3^+^ T cells, activated CD19^+^CD86^+^ B cells, Th1 cells in the spleen and lymph nodes, and Th17 cells in lymph nodes in Sle1.Yaa.*Δ*sNASP mice were increased compared to those in Sle1.Yaa mice. The levels of IFN-*γ* and TNF-*α* in the serum of Sle1.Yaa.*Δ*sNASP mice were higher than those of Sle1.Yaa mice. The above results show that mutant sNASP can interact with different lupus susceptibility genes and promote the disease process of systemic lupus erythematosus.

## 1. Introduction

Systemic lupus erythematosus (SLE) is a highly heterogeneous autoimmune disorder that causes damage to multiple organ systems. The interactions between susceptibility genes and environmental factors play a dominant role and result in an irreversible loss of immunologic self-tolerance [1]. So far, genome-wide association studies (GWAS) have identified more than 100 alleles (SNPs), which increase the risk of developing lupus in humans [2]. However, due to the inherent limitations of human clinical studies, the role of these genes is rarely elucidated. Because the clinical manifestations of spontaneous lupus mice are comparable to those of human SLE patients, various mouse models have been developed to dissect the genetic and cellular mechanisms of SLE [2], as well as to identify and validate therapeutic targets [3].

The murine lupus susceptibility locus Sle2c1 derived from the NZM2410 strain has been proven to promote the proliferation of B1a cells in the abdominal cavity and synergism with lpr mutation in the B6.Sle2.lpr mice, leading to more severe lupus nephritis and marked lymphadenopathy compared to B6.lpr mice [4]. Recently, a variant of somatic nuclear autoantigenic sperm protein (sNASP), which is a chaperone of histones, was identified from the Sle2c1 locus by whole exome sequencing (WES). A nonsynonymous mutation of two adjacent bases on the sNASP gene resulted in changes of the 281 and 282 amino acids in the histone binding region from valine and leucine to isoleucine and phenylalanine. In our previous study, we revealed that the mutant sNASP has a greater affinity to the H4 histone or H3.1/H4 tetramer than the normal sNASP protein *in vitro*, suggesting that the amino acid changes alter its three-dimensional structure and function. To confirm the role of mutant sNASP in Sle2-mediated autoimmune phenotypes, we generated B6.*Δ*sNASP.lpr mice by introgressing the mutant sNASP onto B6.lpr mice and examined evidence of disease at 6 months of age [5]. Our results demonstrate that the variant of sNASP in the B6.lpr strain was responsible for aggravating inflammatory pathology alterations in the kidney and lung and the majority of the cellular dysfunction in the spleen and lymph nodes.

Sle1.Yaa mice contain Sle1 and Y-linked autoimmune accelerator (Yaa) loci and develop a lupus-like disease with splenomegaly and glomerular nephritis in males [6]. Although the Yaa locus translocated from the X chromosome may contain as many as 16 genes, the major candidate gene for causation of the Yaa-associated autoimmune phenotype has been TLR7 [7]. Decreased autoantigen tolerance is part of the manifestation of SLE. Polymorphisms of the SLAM/CD2 gene cluster identified in the Sle1 locus are responsible for the loss of tolerance to the H2A/H2B/DNA subnucleosome antigen [8]. Yaa is highly epistatic with the Sle1 locus, culminating in severe pathology and fatal disease at a relatively young age and release of inflammatory cytokines.

Whether mutant sNASP works synergistically with other lupus genes to promote the inflammatory response and progression is unknown. Therefore, we introduced the mutant sNASP into Sle1.Yaa mice to determine whether it has epistatic product effect with Sle1 or Yaa loci. The synergistic effect between the sNASP mutant and the Sle1.Yaa locus was determined by measuring the changes of lymphocyte subsets in peripheral immune organs, terminal organ damage, levels of serum inflammatory cytokines, and survival time of mice.

## 2. Materials and Method

### 2.1. Mice

Sle1.Yaa mice were purchased from the Jackson Laboratory (Bar Harbor, ME, USA). The Sle1.Yaa.*Δ*sNASP line was derived by breeding female B6.*Δ*sNASP mice to male B6.Sle1.Yaa mice and subsequent intercrossing of progeny. All animals were cared for under experimental protocols approved by the Weifang Medical University Animal Care Committee and housed in a specific pathogen-free (SPF) facility.

### 2.2. Data and Sample Collection

Mice were sacrificed at 6 months after birth, and the spleen, lymph nodes, kidney, and lung were collected. Weight of fresh organs was compared between the two genotypes. Spleen and lymph nodes were ground to prepare single-cell suspension, and cell subsets were determined by flow cytometry. Pathological lesions of the kidney and lung were evaluated after staining. Proteinuria was measured using Coomassie brilliant blue G-250 (Solarbio, Beijing, China) as per the manufacturer's instructions. Bull serum albumin (BSA) serial dilutions were prepared for a standard curve.

### 2.3. Kidney and Lung Histopathology

For histology, tissues of the kidney and right upper lobe lung were fixed in 4% paraformaldehyde and embedded in paraffin, and the sections were stained with hematoxylin and eosin (H&E). In addition, the sections of kidney were also stained with periodic acid Schiff (PAS). Renal and lung lesions were scored in a blind method as the previous reports [9, 10]; glomerular lesions and lung pathology alterations were rated as grades 0-4 from normal to severe as previously described [5]. For immunohistochemistry, kidneys were embedded in an optimal cutting temperature (OCT) compound and stored at −80°C. Frozen sections were fixed in cold acetone at 4°C for 10 min and blocked with 2% newborn bovine serum (NBS) for 10 min. Sections were stained with FITC-conjugated rat anti-mouse C3 (SC-58926, Santa Cruz Biotechnology, Dallas, TX) and IgG*κ* BP-CFL 488 (SC-516176, Santa Cruz Biotechnology, Dallas, TX). All images were obtained by Olympus BX53 fluorescence microscope and a DP80 camera. On average, 20 glomeruli were randomly selected from each sample and semiquantitatively assessed as 0-4 grades according to staining intensity using ImageJ software (NIH).

### 2.4. Cytokine ELISA

Serum concentrations of the cytokines IL-1*β*, IL-6, IL-17, IFN-*γ*, and TNF-*α* were determined by mouse uncoated ELISA kits (Invitrogen, Carlsbad, CA, USA) according to the manufacturer's instructions.

### 2.5. Flow Cytometry

lymphocyte subsets in the spleen and lymph nodes were analyzed by flow cytometry as previously described. In brief, the isolated immunocytes were first blocked with excessive rat anti-mouse CD16/32 antibody (2.4G2) for 30 min at 4°C, then stained with antibodies for 30 min at 4°C, washed and determined by a BD FACSVerse flow cytometer (BD Biosciences, San Jose, CA, USA).The antibodies used for flow cytometry were CD3 (17A2), CD4 (RM), CD8 (53-6.7), CD19 (1D3), CD86 (GL1), CD5 (53-7.3), CXCR5 (2G8), PD-1 (J43), CD1d (1B1), IFN-*γ* (XMG1.2), IL-4 (11B11), IL-17A (TC11-18H10), and CD69 (H1.2F3). All antibodies were purchased from BD Pharmingen (San Jose, CA, USA) or eBioscience (San Diego, CA, USA). For intracellular staining, cells were first stimulated with the leukocyte activation cocktail (BD Biosciences, San Jose, CA, USA) for 4 h, then fixed with the fixation/perm dilution (Invitrogen, Carlsbad, CA, USA) before intracellular staining.

### 2.6. Statistical Analysis

Data analyses and graphs were performed with Prism 5.0 software (GraphPad). Student's paired *t*-test was used for comparison between the two groups. All values were reported as mean ± SEM, and *P* < 0.05 was considered significant.

## 3. Results

### 3.1. The sNASP Variant Aggravates End-Organ Damage in Sle1.Yaa.*Δ*sNASP Mice

**Figure 1:**
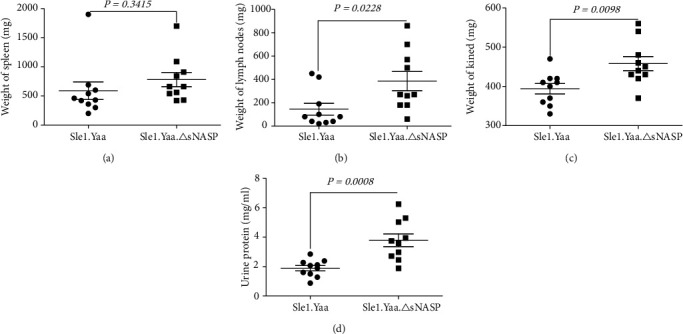
The sNASP variant promotes end-organ damage in Sle1.Yaa mice. The weight of the spleen (a), lymph nodes (b), and kidney (c). Determination of urine protein (d).

To confirm whether the mutant sNASP has epistatic interactions with the Sle1 and Yaa loci, we analyzed the pathological alterations of the Sle1.Yaa.*Δ*sNASP strain compared to the control Sle1.Yaa mice at 6 months of age. The spleen sizes of Sle1.Yaa.*Δ*sNASP were comparable to that of Sle1.Yaa mice(Figure 1(a)), but Sle1.Yaa.*Δ*sNASP mice presented larger pooled lymph nodes (385.04 ± 81.92 mg), about 3 times larger than that of Sle1.Yaa mice (145.02 ± 50.78 mg). Sle1.Yaa.*Δ*sNASP mice also presented larger kidneys than that of Sle1.Yaa mice (Figure 1(c)), which were consistent with the increase of proteinuria in Sle1.Yaa.*Δ*sNASP mice (Figure 1(d)).

**Figure 2:**
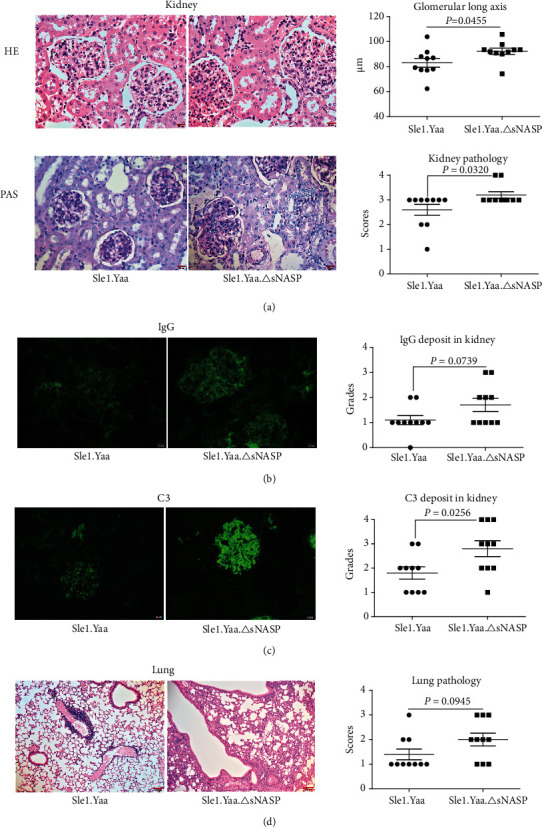
Pathological changes of the kidney and lung in mice. Representative HE-stained and PAS-stained kidney section (×400 magnification) and renal histopathology scores (a). Representative images of mouse IgG (b) and C3 (c) deposit in glomeruli from Sle1.Yaa and Sle1.Yaa.*Δ*sNASP mice (×400 magnification) and their respective fluorescence intensity grades. Representative H&E-stained lung section (×100 magnification) and pulmonary histopathology scores (d).

The pathology of their kidneys and lungs was also examined in our studies and showed more severe end-organ damage in sNASP mutant mice. Glomerular enlargement, mesangial cell proliferation, mesangial expansion, glomerular necrosis, tubular edema, and necrosis were observed in both groups. However, the degree of glomerular endovascular hyperplasia, mesangial cell proliferation, inflammatory cell infiltration, and renal tubular lesions, such as edema and necrosis of renal tubular epithelial cells, was higher than in Sle1.Yaa mice. We measured the long axis of the glomerulus and compared the size of the glomeruli of the two groups of mice. The results showed that the glomeruli of Sle1.Yaa.*Δ*sNASP mice were significantly larger than those of Sle1.Yaa mice (Sle1.Yaa: 83.15 ± 3.485 *μ*m, Sle1.Yaa.*Δ*sNASP: 92.33 ± 2.474 *μ*m, *P* = 0.0455) (Figure 2(a)). Immune complexes in the kidney were detected using the indirect immunofluorescence staining. The Sle1.Yaa.*Δ*sNASP mice showed significantly more C3 deposition in glomeruli than Sle1.Yaa (Figure 2(c)), yet there was no statistical difference for IgG deposition in glomeruli between Sle1.Yaa.*Δ*sNASP and Sle1.Yaa mice (Figure 2(b)). Pathological manifestations of inflammatory cell exudation, alveolar wall thickness, and fibrosis were observed in the lungs of both groups. Sle1.Yaa.*Δ*sNASP mice appeared to have developed more severe lung inflammation and fibrosis; however, no significant statistical difference in the histopathological score was observed (Figure 2(d)).

### 3.2. The sNASP Variant Can Increase the Levels of Serum Inflammatory Cytokines in Sle1.Yaa.*Δ*sNASP Mice

**Figure 3:**
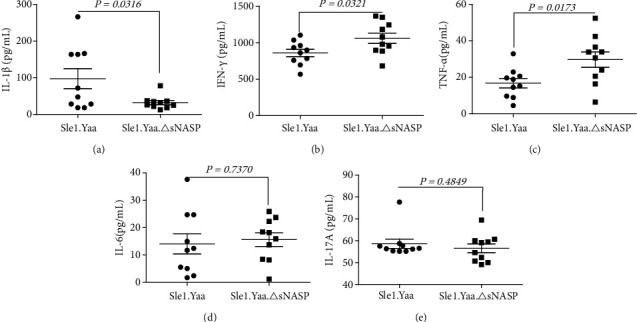
The level of serum inflammatory cytokines in mice. Decreased level of IL-1*β* in Sle1.Yaa.*Δ*sNASP mice compared with Sle1.Yaa mice (a), but the levels of IFN-*γ* (b) and TNF-*α* (c) were increased in Sle1.Yaa.*Δ*sNASP mice compared with Sle1.Yaa mice. The levels of IL-6 (d) and IL-17A (e) had no significant change.

Cytokines play an important role in SLE initiation and progression, so we measured the expression of serum IL-1*β*, IL-6, IL-17, IFN-*γ*, and TNF-*α* using ELISA. Interestingly, IL-1*β* was decreased in Sle1.Yaa.*Δ*sNASP mice (Figure 3(a)), while IFN-*γ* (Figure 3(b)) and TNF-*α* (Figure 3(c)) were increased compared with Sle1.Yaa mice, and there was no difference in the levels of IL-17 (Figure 3(d)) and IL-6 (Figure 3(e)) between the two groups. These results reveal that IFN-*γ* and TNF-*α* may play a major role in the exacerbation of inflammation led by mutant sNASP.

### 3.3. Sle1.Yaa.*Δ*sNASP Mice Developed More Severe Lymphocyte Subset Disorder

**Figure 4:**
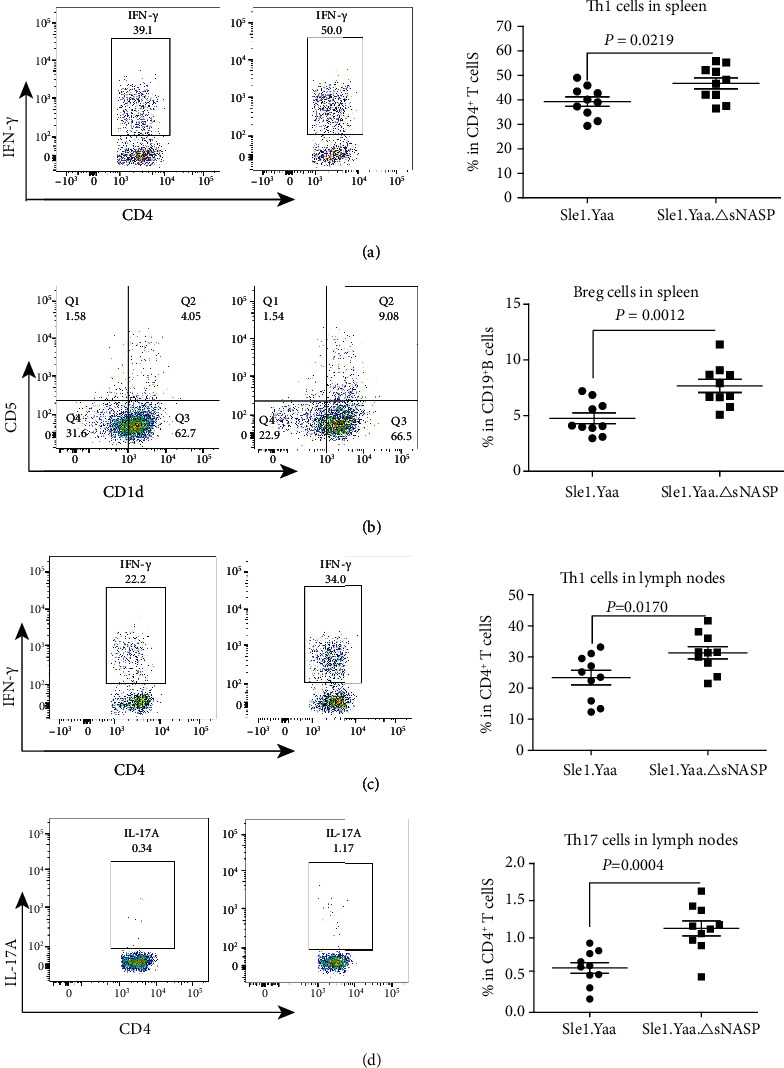
Changes of lymphocyte subsets in spleen and lymph nodes of mice. Representative FACS plots and percentages of Th1 (CD4^+^IFN-*γ*^+^) cells (a) and Breg (CD19^+^CD5^+^CD1d^high^) cells (b) in the spleen, as well as Th1 (CD4^+^IFN-*γ*^+^) cells (c) and Th17 (CD4^+^IL-17A^+^) cells (d) in lymph nodes.

The imbalance and dysfunction of T and B lymphocyte subsets is an important cause of SLE. Therefore, we compared the main lymphocyte subsets in the spleen and lymph nodes between two groups of mice (Table 1). Compared with the Sle1.Yaa mice, the percentages of CD3^+^ T cells, activated CD19^+^CD86^+^ B cells, Th1 (CD4^+^IFN-*γ*^+^) cells (Figure 4(a)), and CD19^+^CD5^+^CD1d^high^ Breg cells (Figure 4(b)) increased significantly while CD19^+^B cells decreased in the spleen of Sle1.Yaa.*Δ*sNASP mice. In the lymph nodes, the percentages of activated CD19^+^CD86^+^ B cells, CD4^+^IFN-*γ*^+^ Th1 cells (Figure 4(c)), and CD4^+^IL-17A^+^ Th17 cells (Figure 4(d)) in the Sle1.Yaa.*Δ*sNASP mice were greater than those in the Sle1.Yaa mice. Other subsets, such as CD3^+^CD4^+^ T cells, CD3^+^CD4^+^ T cells, Tfh, activated CD4^+^CD69^+^ T, and CD4^+^IL-4^+^ Th2 cells, were comparable between Sle1.Yaa.*Δ*sNASP and Sle1.Yaa mice.

### 3.4. Effect of sNASP Gene Mutation on the Survival Rate of Sle1.Yaa Mice

**Figure 5:**
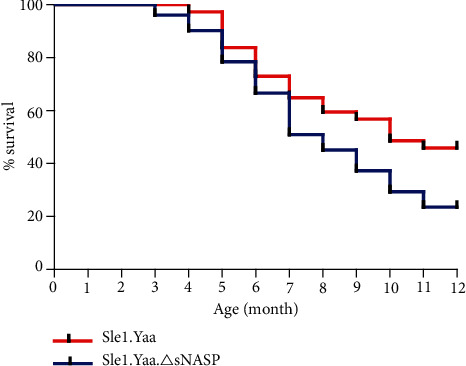
Effect of sNASP gene mutation on the survival rate of Sle1.Yaa mice. Survival was monitored in two groups of Sle1.Yaa (*n* = 37) and Sle1.Yaa.*Δ*sNASP (*n* = 51) mice up to 12 months. A Kaplan-Meir survival analysis was shown. The log-rank (Mantel-Cox) test was applied with a *P* value = 0.0411.

An overall survival trend for Sle1.Yaa.*Δ*sNASP compared to the Sle1.Yaa mice up to 12 months is shown in Figure 5. Kaplan-Meir lifespan analysis indicated a significant *P* value of 0.0411 for overall survival differences. The variant of sNASP decreased the survival rate of Sle1.Yaa mice from 72.973% to 66.667% by month 6, 56.757% to 37.255% by month 9, and 45.946% to 23.529% by month 12 (Figure 5).

## 4. Discussion

Spontaneous mouse lupus models, such as NZB/W F1, NZM2410, MRL/lpr, and BXSB/Yaa, are useful tools for the study of the etiology of the disease. A series of gene alterations found in these models are deemed to be related to lupus, such as the Fas gene in MRL/lpr and TLR7 in BXSB/Yaa mice. In the past decade, three lupus susceptibility genes, CDKN2c, CSF3R, and mutant Skint6, have been identified from the Sle2c1 locus of the NZM2410 strain [4, 5, 10, 11]. Recently, we revealed a novel mutant sNASP in the Sle2c1 locus, which can cooperate with Fas^lpr^ mutation to amplify autoimmunity and greatly exacerbate kidney and lung damage in the B6.lpr strain [5]. Whether mutant sNASP works synergistically with other lupus genes to promote the inflammatory response and progression is unknown. Therefore, we introduced the mutant sNASP into Sle1.Yaa mice to determine whether it has an epistatic product effect with Sle1 or Yaa loci. As predicted, the mutant sNASP further aggravated the immune disorders in Sle1.Yaa.*Δ*sNASP mice and led to a stronger autoimmune response, which was reflected in the hyperplasia of peripheral lymphoid organs, especially lymph nodes.

T cells have been found to play a crucial role in the pathogenesis of SLE [12, 13], among which CD4^+^ T cells are active mediators for the pathogenesis of SLE [13, 14]. Cytokine dysregulation promotes immune dysfunction, and tissue inflammation is one of the hallmarks of SLE [15, 16]. Th1 cells secrete cytokines such as IFN-*γ* and TNF-*α*, which can activate macrophages and inflammatory T lymphocytes. Uncontrolled Th1 activity devotes to a wide range of autoimmune disorders [17, 18]. A host of reports on spontaneous and induced lupus models pointed to the onset of SLE associated with high levels of IFN-*γ* and TNF-*α* [18, 19]. Compared to Sle1.Yaa mice, the proportion of Th1 cells secreting IFN-*γ* in peripheral lymphoid organs in Sle1.Yaa.*Δ*sNASP mice was significantly higher, and the levels of IFN-*γ* and TNF-*α* in the serum were also increased. We hypothesize that the Th1 subset is one of the main targets of the mutant sNASP and contributes to the lupus pathogenesis, and the variant leads to uncontrolled IFN-*γ* and TNF-*α* transcription. In addition to Th1 cell subsets, more and more studies have confirmed that IL-17 plays a momentous role in human SLE patients and murine lupus models [20, 21]. Our results indicated that the proportion of Th17 cells in the lymph nodes of Sle1.Yaa.*Δ*sNASP mice increased significantly and almost twice that of Sle1.Yaa mice. Studies have shown that IL-17 can promote autoreactive B cell proliferation and differentiation and autoantibody production alone or in combination with the B cell activating factor (BAFF) [22]. As a potent proinflammatory cytokine, IL-17 can induce various cells such as epithelial cells and fibroblasts to secrete chemokines and cytokines to mediate tissue damage [23, 24]. Thus, IL-17 may be another contributor among the CD4^+^ T lymphocyte subsets to more end-organ severe glomerular mesangial expansion and pulmonary interstitial fibrosis in sNASP mutant mice. We were surprised to find that there was no difference in the proportion of CD4^+^CD69^+^ T cells in Sle1.Yaa.*Δ*sNASP compared to Sle1.Yaa. The variant not only affects T cell subsets, we also found that the increased CD19^+^CD86^+^ B cells in both the spleen and lymph nodes indicate enhanced activation of B cells in Sle1.Yaa.*Δ*sNASP mice. Interestingly, the proportion of Breg cells in the spleen of Sle1.Yaa.*Δ*sNASP increased slightly than those in Sle1.Yaa mice. Breg cells mainly mediate immunosuppression by secreting IL-10, while IL-10 can affect the development of T helper lymphocytes by inhibiting Th1 cells secreting IFN-*γ* and IL-2 [25]. We consider that the hyperproliferation of Th1 cells disrupts the balance between Th1 and Breg cells and that increased secretion of IFN-*γ* feedback promotes proliferation and differentiation of Breg cells to inhibit Th1 cells [26, 27].

Similar to what has been reported previously in B6.*Δ*NASP.lpr mice, we found that Sle1.Yaa.*Δ*sNASP mice have more severe renal damage than Sle1.Yaa mice. The proteinuria and C3 disposition of Sle1.Yaa.*Δ*sNASP mice were also increased. In previous studies, we have substantiated that the rec1c allele and the variant of sNASP promote the lung inflammation when coexpressed with the Fas^lpr^ mutation [5]. However, in our present study, we observed that the sNASP mutation only slightly exacerbated lung inflammation in Sle1.Yaa.*Δ*sNASP mice. The above results suggest that mutant sNASP seems to only aggravate the existing inflammatory response rather than start a new inflammatory response, because there is already intense renal inflammation in Sle1.Yaa mice. Along with these manifestations, the survival rate of Sle1.Yaa.*Δ*sNASP mice declined significantly compared to that of Sle1.Yaa mice.

How the mutant NASP promotes inflammation has yet to be further elucidated. As a key molecule in chromosome assembly, sNASP plays an important role in the late stage of DNA replication and chromosome folding [28–30]. It binds to H3, H4, and H1, participates in histone transport, and promotes cell proliferation [30, 31]. The sNASP also regulates chromatin accessibility through maintaining histone H3K9me1 [32], and DNA methylation has been shown to affect the function of T cells in lupus mice [33, 34]. Many studies have shown that the sNASP is involved in the chemical modification of histones H3 and H4. After binding the histone H3-H4 heterodimer, the sNASP mediates histone acetyltransferase activity 1 (HAT1) to the carboxyl end of histone H4 and catalyzes the acetylation of lysine at positions 5 and 12 (h4k5ac, h4k12ac). A recent study showed that sNASP can negatively regulate toll-like receptor signaling by binding tumor necrosis factor receptor associated factor 6 (TRAF6) and preventing its ubiquitination in the cytoplasm of the macrophage [35]. As a candidate gene for human SLE [36], TRAF6 has a central role in the nuclear factor NF-*κ*B activation pathway. It regulates inflammation, survival, and activation of multiple immune cell subsets.

In conclusion, the mutant sNASP may participate in the disease process by changing the acetylation modification of the histone, DNA methylation of immune cells, or TRAF6-centered signaling transduction pathway. At least one point, as in our preliminary study, the mutant sNASP enhances the response of macrophages to LPS stimulation and leads to increased release of inflammatory cytokines (data not listed). On the whole, this study further confirmed that the mutant sNASP is a unique lupus susceptibility gene, which can cooperate with different lupus susceptibility genes to aggravate inflammatory response and promote disease progression.

## Figures and Tables

**Table 1:** Effects of NASP variant on splenic and lymph nodes' lymphocyte subsets.

Lymphocyte subsets	Spleen		Lymph nodes	
	Sle1.Yaa (*n* = 10)	Sle1.Yaa*Δ*sNASP (*n* = 10)	Sle1.Yaa (*n* = 10)	Sle1.Yaa*Δ*sNASP (*n* = 10)
CD3^+^	32.12 ± 2.377	39.90 ± 1.661^∗^	48.35 ± 3.245	47.54 ± 3.400
CD19^+^	52.85 ± 2.984	44.01 ± 1.633^∗^	42.73 ± 3.195	42.77 ± 3.306
CD3^+^CD4^+^	66.09 ± 2.272	63.54 ± 1.080	57.11 ± 4.764	61.26 ± 2.177
CD3^+^CD8^+^	19.87 ± 1.715	24.06 ± 0.9000	33.09 ± 4.928	27.39 ± 1.980
CD19^+^CD86^+^	32.95 ± 2.352	52.54 ± 2.678^∗∗^	39.00 ± 3.660	49.88 ± 3.172^∗^
CD4^+^CD69^+^	37.70 ± 5.005	35.86 ± 3.319	28.61 ± 2.023	24.54 ± 4.058
Tfh (CD4^+^CXCR5^+^PD-1^+^)	6.308 ± 1.349	5.210 ± 1.487	2.154 ± 0.2812	2.408 ± 0.5558
Breg (CD19^+^CD5^+^CD1d^high^)	4.762 ± 0.4781	7.664 ± 0.5883^∗∗^	3.133 ± 0.3116	3.892 ± 0.2756
Th1 (CD4^+^IFN-*γ*^+^)	39.29 ± 1.980	46.75 ± 2.217^∗^	23.36 ± 2.333	31.36 ± 2.180^∗^
Th2 (CD4^+^IL-4^+^)	1.193 ± 0.2066	0.9120 ± 0.0969	0.5752 ± 0.1106	0.4593 ± 0.0573
Th17 (CD4^+^IL-17A^+^)	0.4778 ± 0.1230	0.5944 ± 0.0679	0.5990 ± 0.0725	1.130 ± 0.1004^∗∗^

Results are mean ± SEM; ^∗^*P* < 0.05 vs. Sle1.Yaa mice; ^∗∗^*P* < 0.01 vs. Sle1.Yaa mice.

## Data Availability

The data used to support the findings of this study are included within the article.

## References

[1] Shaikh M. F., Jordan N., D'Cruz D. P. (2017). Systemic lupus erythematosus. *Clinical Medicine (London, England)*.

[2] Ju J. Y., Xu Z. W. (2021). Potential genetic basis of B cell hyperactivation in murine lupus models. *Lupus*.

[3] Datta S. K. (2021). Harnessing tolerogenic histone peptide epitopes from nucleosomes for selective down-regulation of pathogenic autoimmune response in lupus (past, present, and future). *Frontiers in Immunology*.

[4] Morel L., Rudofsky U. H., Longmate J. A., Schiffenbauer J., Wakeland E. K. (1994). Polygenic control of susceptibility to murine systemic lupus erythematosus. *Immunity*.

[5] Ju J., Xu J., Zhu Y., Fu X., Morel L., Xu Z. (2019). A variant of the histone-binding protein sNASP contributes to mouse lupus. *Frontiers in Immunology*.

[6] Yoachim S. D., Nuxoll J. S., Bynoté K. K., Gould K. A. (2015). Estrogen receptor alpha signaling promotes _Sle1_ -induced loss of tolerance and immune cell activation and is responsible for sex bias in B6. _Sle1_ congenic mice. *Clinical Immunology*.

[7] Subramanian S., Tus K., Li Q. Z. (2006). A Tlr7 translocation accelerates systemic autoimmunity in murine lupus. *Proceedings of the National Academy of Sciences of the United States of America*.

[8] Mohan C., Alas E., Morel L., Yang P., Wakeland E. K. (1998). Genetic dissection of SLE pathogenesis. Sle1 on murine chromosome 1 leads to a selective loss of tolerance to H2A/H2B/DNA subnucleosomes. *The Journal of clinical investigation*.

[9] Xu Z., Cuda C. M., Croker B. P., Morel L. (2011). The NZM2410-derived lupus susceptibility locus Sle2c1 increases Th17 polarization and induces nephritis in fas-deficient mice. *Arthritis and Rheumatism*.

[10] Xu Z., Xu J., Ju J., Morel L. (2017). A Skint6 allele potentially contributes to mouse lupus. *Genes and Immunity*.

[11] Xu Z., Potula H. H., Vallurupalli A. (2011). Cyclin-dependent kinase InhibitorCdkn2cRegulates B cell homeostasis and function in the NZM2410-derived murine lupus susceptibility LocusSle2c1. *Journal of Immunology*.

[12] Chen P. M., Tsokos G. C. (2021). T cell abnormalities in the pathogenesis of systemic lupus erythematosus: an update. *Current Rheumatology Reports*.

[13] Sharabi A., Tsokos G. C. (2020). T cell metabolism: new insights in systemic lupus erythematosus pathogenesis and therapy. *Nature Reviews Rheumatology*.

[14] Li M., Yang C., Wang Y. (2020). The expression of P2X7 receptor on Th1, Th17, and regulatory T cells in patients with systemic lupus erythematosus or rheumatoid arthritis and its correlations with active disease. *Journal of Immunology*.

[15] Ohl K., Tenbrock K. (2011). Inflammatory cytokines in systemic lupus erythematosus. *Journal of Biomedicine & Biotechnology*.

[16] Chasset F., Arnaud L. (2018). Targeting interferons and their pathways in systemic lupus erythematosus. *Autoimmunity Reviews*.

[17] Fava A., Buyon J., Mohan C. (2020). Integrated urine proteomics and renal single-cell genomics identify an IFN-*γ* response gradient in lupus nephritis. *JCI Insight*.

[18] Theofilopoulos A. N., Koundouris S., Kono D. H., Lawson B. R. (2001). The role of IFN-gamma in systemic lupus erythematosus: a challenge to the Th1/Th2 paradigm in autoimmunity. *Arthritis Research*.

[19] Pollard K. M., Cauvi D. M., Toomey C. B., Morris K. V., Kono D. H. (2013). Interferon-*γ* and systemic autoimmunity. *Discovery Medicine*.

[20] Koga T., Ichinose K., Kawakami A., Tsokos G. C. (2019). The role of IL-17 in systemic lupus erythematosus and its potential as a therapeutic target. *Expert Review of Clinical Immunology*.

[21] Clarke J. (2020). IL-17 sustains plasma cells in SLE. *Nature Reviews Rheumatology*.

[22] Doreau A., Belot A., Bastid J. (2009). Interleukin 17 acts in synergy with B cell-activating factor to influence B cell biology and the pathophysiology of systemic lupus erythematosus. *Nature Immunology*.

[23] Gaffen S. L. (2008). An overview of IL-17 function and signaling. *Cytokine*.

[24] Onishi R. M., Gaffen S. L. (2010). Interleukin-17 and its target genes: mechanisms of interleukin-17 function in disease. *Immunology*.

[25] Oleszycka E., McCluskey S., Sharp F. A. (2018). The vaccine adjuvant alum promotes IL-10 production that suppresses Th1 responses. *European Journal of Immunology*.

[26] Ma K., du W., Wang X. (2019). Multiple functions of B cells in the pathogenesis of systemic lupus erythematosus. *International Journal of Molecular Sciences*.

[27] Rosser E. C., Mauri C. (2015). Regulatory B cells: origin, phenotype, and function. *Immunity*.

[28] Richardson R. T., Alekseev O. M., Grossman G. (2006). Nuclear Autoantigenic Sperm Protein (NASP), a Linker Histone Chaperone That Is Required for Cell Proliferation∗. *Journal of Biological Chemistry*.

[29] Finn R. M., Browne K., Hodgson K. C., Ausió J. (2008). sNASP, a Histone H1-Specific Eukaryotic Chaperone Dimer that Facilitates Chromatin Assembly. *Biophysical Journal*.

[30] Apta-Smith M. J., Hernandez-Fernaud J. R., Bowman A. J. (2018). Evidence for the nuclear import of histones H3.1 and H4 as monomers. *The EMBO Journal*.

[31] Cook A. J., Gurard-Levin Z. A., Vassias I., Almouzni G. (2011). A specific function for the histone chaperone NASP to fine-tune a reservoir of soluble H3-H4 in the histone supply chain. *Molecular Cell*.

[32] Kang X., Feng Y., Gan Z. (2018). NASP antagonize chromatin accessibility through maintaining histone H3K9me1 in hepatocellular carcinoma. *Biochimica et Biophysica Acta - Molecular Basis of Disease*.

[33] Li H., Tsokos M. G., Bickerton S. (2018). Precision DNA demethylation ameliorates disease in lupus-prone mice. *JCI Insight*.

[34] Vecellio M., Wu H., Lu Q., Selmi C. (2021). The multifaceted functional role of DNA methylation in immune-mediated rheumatic diseases. *Clinical Rheumatology*.

[35] Yang F. M., Zuo Y., Zhou W. (2018). sNASP inhibits TLR signaling to regulate immune response in sepsis. *Journal of Clinical Investigation*.

[36] Namjou B., Choi C. B., Harley I. T. (2012). Evaluation ofTRAF6in a large multiancestral lupus cohort. *Arthritis and Rheumatism*.

